# Transcriptomic and proteomic profiling revealed reprogramming of carbon metabolism in acetate-grown human pathogen *Candida glabrata*

**DOI:** 10.1186/s12929-020-00700-8

**Published:** 2021-01-02

**Authors:** Shu Yih Chew, Alistair J. P. Brown, Benjamin Yii Chung Lau, Yoke Kqueen Cheah, Kok Lian Ho, Doblin Sandai, Hassan Yahaya, Leslie Thian Lung Than

**Affiliations:** 1 Department of Medical Microbiology, Faculty of Medicine and Health Sciences, Universiti Putra Malaysia, 43400 Serdang, Selangor Malaysia; 2 MRC Centre for Medical Mycology, University of Exeter, Geoffrey Pope Building, Stocker Road, Exeter, EX4 4QD UK; 3 Proteomics and Metabolomics (PROMET) Group, Malaysian Palm Oil Board, Bandar Baru Bangi, 43000 Kajang, Selangor Malaysia; 4 Department of Biomedical Sciences, Faculty of Medicine and Health Sciences, Universiti Putra Malaysia, 43400 Serdang, Selangor Malaysia; 5 Department of Pathology, Faculty of Medicine and Health Sciences, Universiti Putra Malaysia, 43400 Serdang, Selangor Malaysia; 6 Infectomics Cluster, Advanced Medical and Dental Institute, Universiti Sains Malaysia, 13200 Kepala Batas, Pulau Pinang Malaysia; 7 Department of Medical Laboratory Science, Faculty of Allied Health Sciences, Bayero University, Kano, Nigeria

**Keywords:** *Candida*, *Candida glabrata*, Acetate, Carbon metabolism, Transcriptomic, Proteomic, RNA-sequencing, Liquid chromatography tandem-mass spectrometry

## Abstract

**Background:**

Emergence of *Candida glabrata*, which causes potential life-threatening invasive candidiasis, has been widely associated with high morbidity and mortality. In order to cause disease in vivo, a robust and highly efficient metabolic adaptation is crucial for the survival of this fungal pathogen in human host. In fact, reprogramming of the carbon metabolism is believed to be indispensable for phagocytosed *C. glabrata* within glucose deprivation condition during infection.

**Methods:**

In this study, the metabolic responses of *C. glabrata* under acetate growth condition was explored using high-throughput transcriptomic and proteomic approaches.

**Results:**

Collectively, a total of 1482 transcripts (26.96%) and 242 proteins (24.69%) were significantly up- or down-regulated. Both transcriptome and proteome data revealed that the regulation of alternative carbon metabolism in *C. glabrata* resembled other fungal pathogens such as *Candida albicans* and *Cryptococcus neoformans*, with up-regulation of many proteins and transcripts from the glyoxylate cycle and gluconeogenesis, namely isocitrate lyase (*ICL1*), malate synthase (*MLS1*), phosphoenolpyruvate carboxykinase (*PCK1*) and fructose 1,6-biphosphatase (*FBP1*). In the absence of glucose, *C. glabrata* shifted its metabolism from glucose catabolism to anabolism of glucose intermediates from the available carbon source. This observation essentially suggests that the glyoxylate cycle and gluconeogenesis are potentially critical for the survival of phagocytosed *C. glabrata* within the glucose-deficient macrophages.

**Conclusion:**

Here, we presented the first global metabolic responses of *C. glabrata* to alternative carbon source using transcriptomic and proteomic approaches. These findings implicated that reprogramming of the alternative carbon metabolism during glucose deprivation could enhance the survival and persistence of *C. glabrata* within the host.

## Introduction

*Candida glabrata,* an opportunistic human pathogen has become one of the most common etiological agents of invasive candidiasis caused by non-*Candida albicans Candida* (NCAC) species [1–3]. Multiple studies have underlined the essential role of carbon metabolism in the survival of pathogenic *Candida* species [4–7]. These studies suggest that *Candida* species must be able to utilize a wide range of alternative carbon sources such as lactate and acetate within human host niches. In fact, *C. glabrata* has been reported to be able to assimilate various alternative carbon sources from diverse anatomical sites, including intestinal and vaginal microenvironments [8, 9].

It is evident that fungal metabolism, particularly carbon metabolism is of primary importance to human fungal pathogens. In fact, carbon metabolism also affects multiple physiological, immunological and pathogenic attributes of *Candida* species in glucose-deficient condition [10–12]. In *C. albicans*, lactate triggers cell wall remodelling that changes the biophysical features and architecture of the fungal cell wall. Furthermore, alteration of these cell wall (and other) properties also increases the stress resistance of *C. albicans*, including resistance to antifungal drugs, osmotic stress, oxidative stress and cell wall stresses [10]. In contrast, addition of acetic acid to glucose-grown *C. glabrata* renders the cells to be more sensitive to fluconazole treatment, and these cells are also more susceptible to phagocytosis killing by macrophages [12]. Previously, we have demonstrated that alternative carbon sources also induce physiological changes related to the pathogenicity of *C. glabrata* [13]. These include changes in planktonic and biofilm growth, cell wall architecture, oxidative stress resistance and antifungal susceptibility.

The “complete” genome sequence of *C. glabrata* was reported in 2004 [14] and this has proven to be useful in many applications such as transcriptional profiling. Investigations on the global gene expression patterns of fungal pathogens under relevant conditions can potentially assist in the understanding of fungal pathogenesis [15]. Transcriptional analysis of *C. glabrata* following macrophage and neutrophil engulfment revealed a massive reprogramming response that mirrors the transcriptional landscapes of engulfed *C. albicans* cells [4, 5, 15]. Metabolic genes involved in gluconeogenic growth such as genes from gluconeogenesis, the glyoxylate cycle and fatty acid β-oxidation were all significantly up-regulated. In addition, Rai et al. also reported that, during macrophage engulfment, *C. glabrata* mainly utilizes intermediates from fatty acid degradation through the glyoxylate cycle and gluconeogenesis [7]. Given the fact that alternative carbon sources are relevant in vivo, deciphering the transcriptional and proteomic landscapes of *C. glabrata* in response to these carbon sources could be a key to understand the role of fungal metabolism in shaping the physiological behaviour of *C. glabrata*.

The arrival of high-throughput next generation sequencing (NGS) technology has paved the way for advanced research that was not possible over a decade ago [16]. In fact, RNA sequencing has been used extensively in understanding of biological and physiological processes of many fungal species [17–20]. In addition, this approach to genome-wide transcript quantification is also recognized to be superior over other transcriptomic methods [21]. As gene expression is also regulated by post-transcriptional modification and translation processes, additional proteomic analysis is also imperative for complete comprehension of the molecular mechanisms [22]. Label-free quantitative proteomic is reproducible and cost effective [23]. This technique is also able to improve sequence coverage and detection of differentially expressed proteins (DEPs) more consistently, compared to the isobaric tagging for relative and absolute quantification (iTRAQ)-based method [24, 25].

Integrating transcriptomic and proteomic analyses is essential for a more complete understanding of the underlying gene regulation, molecular mechanisms and cellular processes of *C. glabrata* in the presence of physiological relevant alternative carbon source [26]. To achieve this goal, we examined the transcriptional response of *C. glabrata* grown in media containing acetate as the alternative carbon source by using the high-throughput RNA sequencing. In addition, we performed label-free quantitative proteomic analysis using the liquid chromatography–mass spectrometry. It was anticipated that the comprehensive overview provided by these transcriptome and proteome data might shed light on the metabolic regulation of *C. glabrata*, particularly under conditions of glucose deprivation.

## Materials and methods

### Strain and growth conditions

*Candida glabrata* ATCC 2001 (American Type Culture Collection, USA) was used throughout this study. Standard culture media were used, including YPD (Becton, Dickinson and Company, USA): yeast extract (1%, w/v), peptone (2%, w/v), glucose (2%, w/v), agar (1.5%, w/v) and YNB without amino acids (Becton, Dickinson and Company, USA): yeast nitrogen base (0.67%, w/v), ammonium sulfate (0.5%, w/v). Synthetic complete (SC) media were prepared with YNB without amino acids, supplemented with complete supplement mixture (0.2%, w/v) (Formedium, UK), glucose (2%, w/v) or acetate (2%, w/v) (Sigma-Aldrich, USA) as the sole carbon source.

### RNA extraction

Briefly, an overnight culture of *C. glabrata* ATCC 2001 in YPD was washed twice with PBS, pH 7.4, resuspended in fresh YPD (OD_600nm_ of 0.1) and regrown to mid-exponential phase (OD_600nm_ of 0.5). The cell suspension was harvested, washed and resuspended in SC media with 2% (w/v) glucose or 2% (w/v) acetate. Following incubation for 2 h at 37 °C, *C. glabrata* cells were harvested, washed and RNA was extracted using a hot acidic phenol extraction method as previously described [27]. RNA purity and concentration were determined by NanoDrop 1000 spectrophotometer (Thermo Fisher Scientific, USA) and Qubit 2.0 Fluorometer (Thermo Fisher Scientific, USA), respectively. RNA integrity was assessed by Bioanalyzer 2100 using RNA 6000 Nano Kit (Agilent Technologies, USA).

### Library construction and RNA-sequencing

Library construction was carried out using TruSeq RNA Library Prep Kit v2 (Illumina, USA) following manufacturer’s recommendations. Briefly, 200 ng of each RNA sample was purified by oligo-dT beads, and poly(A)-containing mRNA was fragmented into small pieces prior to cDNA synthesis. First strand cDNA was generated by First Strand Master Mix and SuperScript II Reverse Transcriptase (Thermo Fisher Scientific, USA), and incubated at 25 °C for 10 min; 42 °C for 50 min; 70 °C for 15 min. Subsequently, Second Strand Master Mix was added to synthesize the second strand cDNA and incubated at 16 °C for 1 h. Purified fragmented cDNA was combined with End Repair Mix and incubated at 30 °C for 30 min. The end-paired DNA was then purified with AMPure XP^®^ Beads (Beckman Coulter, USA) and incubated with A-Tailing Mix at 37 °C for 30 min. Adenylated 3′ ends DNA was combined with RNA Index Adapter and Ligation Mix, then the ligation reaction was incubated at 30 °C for 10 min. The end-paired DNA was purified with AMPure XP^®^ Beads. Finally, PCR enrichment was performed to enrich the adaptor ligated DNA and the PCR products were purified with AMPure XP^®^ Beads. The library average molecule length was determined by Bioanalyzer 2100 using DNA 1000 Kit (Agilent Technologies, USA), and the library was quantified via real-time PCR. The final library was amplified within the flow cell on the cBot instrument (Illumina, USA) for cluster generation using HiSeq^®^ 4000 PE Cluster Kit (Illumina, USA). Finally, the clustered flow cell was loaded onto the HiSeq 4000 Sequencing System (Illumina, USA) for paired-end sequencing using HiSeq^®^ 4000 SBS Kit (Illumina, USA). In total, the experiments were performed in triplicates using *C. glabrata* grown in two different growth conditions (glucose and acetate).

### Reads mapping and differential expression analysis

Quality of the raw sequencing reads was accessed by FastQC v0.10.1. Prior to reads mapping, low quality reads and bases (quality value < 30 and length size < 50 bp), ambiguous bases and artefacts were removed by FastX toolkit v0.0.13.2. Orphan reads obtained after the trimming and filtering were also removed. Clean reads were mapped to the reference genome of *C. glabrata* CBS 138 (GCF_000002545.3). The reads mapping was carried out using TopHat v2.0.12 [28], which used Bowtie2 tool as algorithmic core, by allowing up to two reads mismatches. The expression of genes annotated in reference genome were quantified using Cufflinks v2.2.1. Differentially expressed genes between glucose-grown *C. glabrata* (control) and acetate-grown *C. glabrata* were analyzed by Cuffdiff from the Cufflinks package [29]. The coverage of reads mapped was translated to expression value which represented in fragments per kilobase of transcript per million mapped fragments (FPKM) [29]. Statistically significant DEGs were called with arbitrary cut-off values of log2 fold change ≥ 1.5 and q value of ≤ 0.05, with false discovery rate (FDR) less than 5%. RNA-sequencing data are available at European Nucleotide Archive (ENA) [30] under the study accession number PRJEB33880.

### Functional annotation and enrichment analysis

Functional annotation and enrichment analyses of DEGs were performed using Database for Annotation, Visualization and Integrated Discovery (DAVID) Bioinformatics Resource 6.8 tool (https://david.ncifcrf.gov/) [31] with DAVID-defined default annotation categories. Enrichment analysis of Gene ontology (GO) was performed to identify and categorize enriched biological process, molecular function and cellular component. In addition, DEGs were also searched against the Kyoto Encyclopedia of Genes and Genomes (KEGG) database to detect significantly enriched pathways.

### Validation of RNA-sequencing data

Validation of RNA sequencing data was performed by quantification of gene expression via quantitative real-time PCR (qPCR). Total RNA was reversibly transcribed to cDNA using Maxima H Minus First Strand cDNA Synthesis Kit (Thermo Scientific, USA), followed by PCR amplification with SensiFAST SYBR^®^ No-ROX Kit (Bioline, UK). Fold changes in relative gene expression were determined by the changes in cycle threshold (CT) values and data obtained were normalized against the β-actin transcript, *ACT1*. Relative fold change was calculated using delta-delta CT method (2^−∆∆CT^). Primers used were described in Additional file 1: Table S1. Primers used in this study were designed by using the free online Primer-BLAST software from National Centre for Biotechnology Information (NCBI). All primers were synthesized commercially by Integrated DNA Technologies, USA.

### Protein extraction

Protein extraction was performed as previously described with slight modification [32]. An overnight cultures of *C. glabrata* ATCC 2001 in YPD was harvested and washed twice with PBS, pH 7.4 before resuspended into fresh SC media (OD_600nm_ of 0.1) supplemented with 2% (w/v) glucose or 2% (w/v) acetate. Following incubation for 24 h at 37 °C, the *C. glabrata* cells were centrifuged at 4000×*g* for 5 min and resuspended in 1 ml of lysis buffer consist of 7 M urea (Bio-Rad Laboratories, USA), 4% (w/v) 3-[(3-cholamidopropyl)dimethylammonio]-1-propanesulfonate (CHAPS) (Bio-Rad Laboratories, USA), 0.4% (w/v) dithiothreitol (DTT) (Bio-Rad Laboratories, USA) and 1 tablet of protease inhibitor cocktail (Roche Diagnostics GmbH, Germany). The cell suspension was mixed with equal volume of 0.5 mm glass beads (BDH Chemicals, VMR, USA) and disrupted mechanically with FastPrep-24™ instrument (MP Biomedicals, USA) at the speed of 6 M/s for 2 cycles of 30 s with short ice-cold incubations in between. Following centrifugation at 3000×*g* for 5 min, proteins in the supernatant were precipitated by adding five volumes of 0.1 M ice-cold ammonium acetate in methanol (Merck, Germany) and incubated overnight at − 20 °C. The protein pellet was collected by centrifugation at 15,000×*g* for 15 min at 4 °C and washed twice with ice-cold 80% (v/v) acetone (Merck, Germany). Finally, the protein pellet was air-dried for 5 min and resuspended in 50 mM ammonium bicarbonate (Sigma-Aldrich, USA) containing 1 M urea. The protein concentrations in each sample was quantified with Pierce 660 nm protein assay reagent (Thermo Fisher Scientific, USA).

### In-solution protein digestion

In-solution protein digestion was performed according to a modified procedure previously described [33]. Prior to protein digestion, protein samples were reduced with 100 mM tris(2-carboxyethyl) phosphine (TCEP) (Sigma-Aldrich, USA) in 50 mM ammonium bicarbonate for 1 h at 60 °C, followed by alkylation using 200 mM iodoacetamide (Bio-Rad Laboratories, USA) in 50 mM ammonium bicarbonate for 45 min at room temperature. Subsequently, 1% (w/v) sodium deoxycholate (Sigma-Aldrich, USA) in 5 mM ammonium bicarbonate was added and the protein samples were incubated for 10 min at 37 °C. Protein samples were mixed with modified sequencing grade trypsin (Promega, USA) in a ratio of 1 µg trypsin to 50 µg protein, 10% (v/v) acetonitrile (Merck, Germany) in 5 mM ammonium bicarbonate, was added before the mixture was incubated for 16 h at 37 °C. Sodium deoxycholate was removed by incubating the peptide samples with 0.5% (v/v) formic acid (Fisher Scientific, USA) for 45 min at 37 °C. Peptide samples were centrifuged at 14,000×*g* for 15 min and dried in a vacuum concentrator (Concentrator Plus, Eppendorf, Germany). Dried peptide pellet was resuspended in 100 µl of 0.1% (v/v) formic acid. Acetonitrile-washed and methanol-conditioned Empore solid phase extraction (SPE) C18 disks (3 M Science, USA) was added to the resuspended pellet and incubated for 3 h at room temperature with mild agitation. Following 3 h of incubation, C18 disks were removed from the peptide samples and the bound peptides were eluted by using 100 µl of elution buffer (50% acetonitrile in 0.1% (v/v) formic acid) for 30 min. The eluent was transferred to a clean microcentrifuge tube and dried with the vacuum concentrator.

### Liquid chromatography tandem-mass spectrometry

For mass spectrometric analysis, peptide digests were reconstituted in 30 µl of 0.1% (v/v) formic acid and 5% (v/v) acetonitrile prior to sample loading. A nano-flow ultra-high-performance liquid chromatography (UHPLC) instrument (EASY-nLC 1200 System, Thermo Fisher Scientific, USA) coupled online to a Q Exactive Plus Quadrupole-Orbitrap mass spectrometer (Thermo Fisher Scientific, USA) with a Nanospray Flex ion source was used for analysis. In total, the experiments were performed in duplicates using *C. glabrata* grown in two different growth conditions (glucose and acetate). Chromatography column used for separation was Acclaim PepMap 100 C18 (Thermo Fisher Scientific, USA). Approximately 2 µg of the peptide mixture was loaded onto the reversed phase column (15 cm long, 75 μm inner diameter) and separated with a linear gradient of 5–50% Buffer B (80% (v/v) acetonitrile and 0.1% (v/v) formic acid) at a flow rate of 300 nl/min for 90 min.

The spray voltage was set to 1.96 kV, funnel radial force level at 50, and heated capillary at 275 ºC. The Q Exactive Plus was configured for data-dependent acquisition using the full MS/DD-MS/MS setup. Top15 method dynamically chose 15 of the most abundant precursor ions from the survey scan for high-energy collisional dissociation (HCD) fragmentation. Full MS resolution was set to 70,000 at *m*/*z* 200 and full MS automatic gain control (AGC) target was 3e6 with a maximum injection time of 100 ms. Mass range was set to 310–1800 *m/z*. Resolution for HCD spectra was set to 17,500 at *m*/*z* 200. AGC target value for fragment spectra was set at 1e5, intensity threshold was kept at 8.3e4 and maximum injection time was set to 60 ms. Isolation width was set at 0.7 *m*/*z*. Normalised collision energy was set at 27%. Peptide match was set to preferred, and isotope exclusion was on. All data were acquired in profile mode using positive polarity.

### Data processing and protein identification

Mass spectrometry data obtained were analyzed with the Proteome Discoverer version 2.2 (Thermo Fisher Scientific, USA) using the integrated SEQUEST search engine against the *Candida* UniProt protein database (September 2018 release, 231,653 sequence entries). Trypsin was specified as the enzyme, cleaving after lysine and arginine residues and allowing up to two missed cleavages. Carbamidomethylation of cysteine was set as fixed modification and *N*-terminal protein acetylation, methionine oxidation, asparagine and glutamine deamidation as variable or dynamic modifications. Proteome Discoverer was used to score peptides for identification based on a search with an initial allowed mass deviation of the precursor ion of up to 10 ppm. The allowed fragment mass deviation was 0.02 Da. The FDR was set to 0.01 for proteins and peptides, which had to have a minimum length of 6 amino acids. To further reduce the false peptide identifications, percolator algorithm was applied to discriminate between the correct and incorrect peptide spectrum matches based on probability and q value. All subsequent statistical analysis was performed using the protein and peptide level data using Proteome Discoverer. Peak intensities were normalized and subjected to the principal component analysis. Volcano plot was used to visualize the expression changes. The plot was derived from the protein/peptide abundance ratios and p values. The mass spectrometry proteomics data have been deposited to the ProteomeXchange Consortium via the PRIDE [34] partner repository with the dataset identifier PXD014916 and 10.6019/PXD014916.

## Results

### Transcriptomic landscape of acetate-grown *C. glabrata*

Comparative RNA sequencing was employed to identify the differential gene expression between acetate-grown *C. glabrata* and glucose-grown *C. glabrata* (control). In general, approximately 65 million raw reads were obtained from transcriptome for all six samples (each growth condition in triplicate). Following the process of quality filtering, over 98% of the sequencing data were retained and less than 0.5% reads were removed as orphan reads (Table 1). High quality cleaned reads were then mapped to the reference genome of *C. glabrata* CBS 138, and high percentage of the reads (93%) were uniquely mapped to the selected genome. In addition, high correlations were observed among the three biological replicates for the glucose-grown *C. glabrata* (0.94 < r^2^ < 0.95) and acetate-grown *C. glabrata* (0.95 < r^2^ < 0.96) (Additional file 1: Table S2).

**Table 1:** Summary of the transcriptome data and genome mapping from glucose- and acetate-grown *C. glabrata* samples

Sample group	Glucose-grown *C. glabrata*			Acetate-grown *C. glabrata*		
	Glu 1	Glu 2	Glu 3	Ace 1	Ace 2	Ace 3
Total raw reads	11,167,982	11,067,988	11,108,556	11,026,716	10,713,412	10,502,714
Total clean reads	11,069,487	10,968,492	11,012,892	10,927,921	10,615,974	10,400,162
Paired reads	11,035,394	10,934,808	10,978,978	10,894,900	10,583,306	10,368,454
Orphan reads	34,093	33,684	33,914	33,021	32,668	31,708
GC content (%)	44	45	45	45	43	44
Reads uniquely mapped	10,260,577 (93.0%)	10,186,208 (93.1%)	10,292,990 (93.7%)	10,177,516 (93.4%)	9,997,771 (94.5%)	9,733,441 (93.9%)
Reads multiply mapped	380,256 (3.4%)	325,515 (3.0%)	315,263 (2.9%)	163,869 (1.5%)	137,062 (1.3%)	147,681 (1.4%)
Reads unmapped	394,561 (3.6%)	423,085 (3.9%)	370,725 (3.4%)	553,515 (5.1%)	448,473 (4.2%)	487,332 (4.7%)

The transcriptome of the acetate-grown *C. glabrata* was compared to glucose-grown *C. glabrata* to calculate the differential gene expression. A cut-off of 1.5-fold change and q value < 0.05 were applied to determine the statistically significant up- and down-regulated genes. Overall, approximately 93% out of 5498 genes were found to be reliably expressed in both conditions, among which 1482 (26.96%) were significantly expressed in acetate-grown *C. glabrata* compared to glucose. Among these 1482 DEGs, 744 (13.53%) were significantly up-regulated and 732 (13.31%) were significantly down-regulated.

### Verification of RNA-sequencing data

**Fig. 1:**
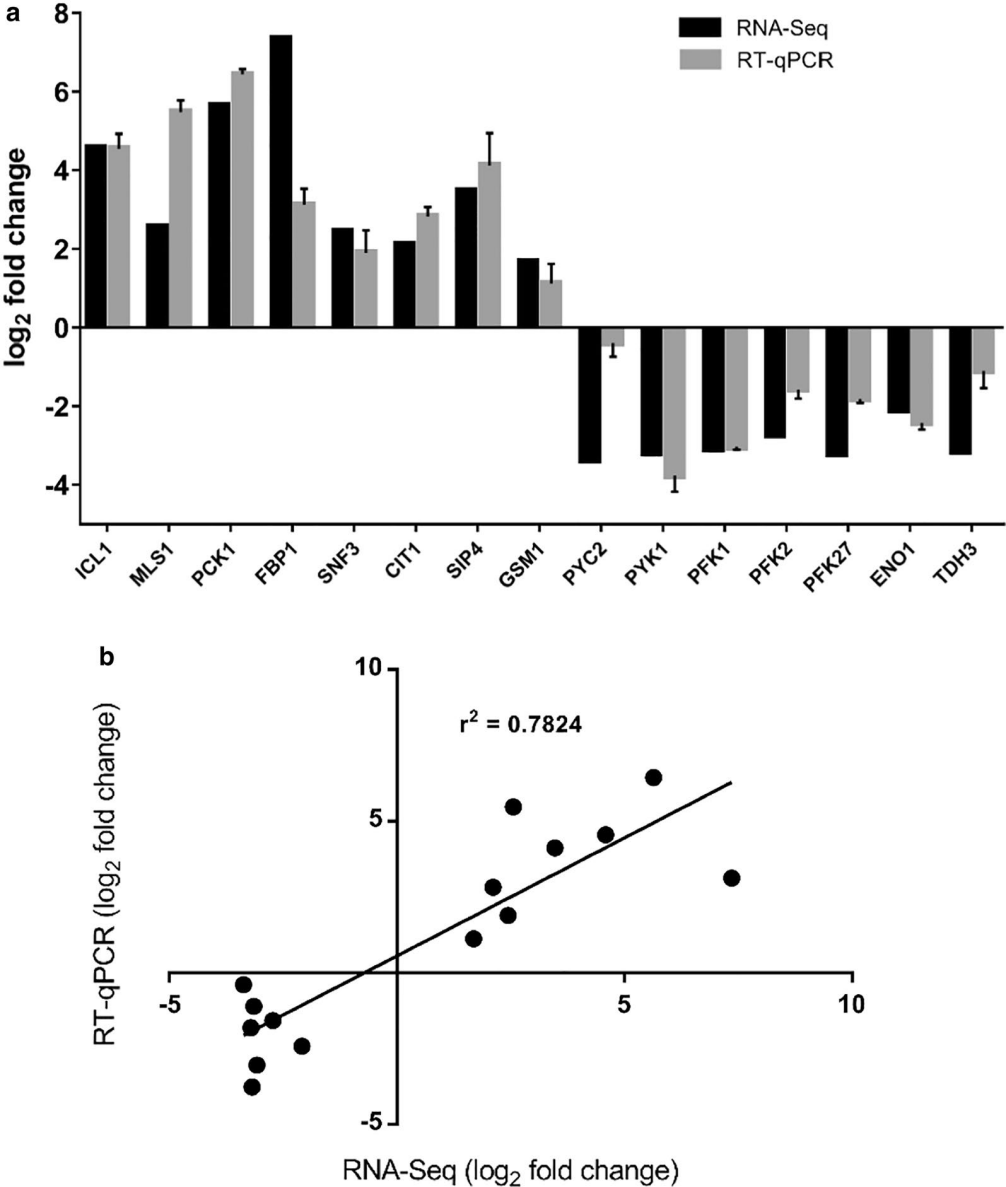
Verification of the RNA sequencing data. **a** Comparison of gene expression levels of 15 DEGs between RNA sequencing and RT-qPCR analyses. **b** Correlation between gene expression levels of 15 DEGs obtained from RNA sequencing and RT-qPCR analyses. Statistically significant Pearson correlation (r^2^ = 0.7824) was observed between the two quantitative methods

Quantitative real-time PCR was performed to test the validity of the results obtained from the RNA sequencing analysis. Fifteen DEGs were chosen for qPCR analysis, which included eight up-regulated genes: isocitrate lyase (*ICL1*), malate synthase (*MLS1*), phosphoenolpyruvate carboxykinase (*PCK1*), fructose 1,6-bisphosphatase (*FBP1*), putative plasma membrane high affinity glucose sensor (*SNF3*), citrate synthase (*CIT1*), C6 zinc cluster transcriptional activator (*SIP4*), putative zinc cluster protein (*GSM1*) and seven down-regulated genes: pyruvate carboxylase (*PYC2*), pyruvate kinase (*PYK1*), phosphofructokinase (*PFK1*), 6-phosphofructokinase (*PFK2*), 6-phosphofructo-2-kinase (*PFK27*), enolase (*ENO1*), glyceraldehyde-3-phosphate dehydrogenase (*TDH3*) in the acetate-grown cells, relative to the control glucose-grown cells. Expression of all DEGs obtained from qPCR analysis were in agreement with results from the RNA sequencing analysis (Fig. 1a). Although the correlation was not so strong on *PYC2*, both RNA sequencing and qPCR data still indicated this gene as down-regulated. Results from RNA sequencing analysis and qPCR also demonstrated significant Pearson correlations (r^2^ = 0.7824), further confirming the reliability of the transcriptome data (Fig. 1b).

### Functional enrichment of the DEGs

Functional enrichment analysis was performed on up- and down-regulated DEGs to detect significant enriched GO and KEGG pathways according to DAVID Bioinformatics Resource 6.8. A total of 744 up-regulated and 732 down-regulated DEGs (in the acetate-, compared to the control glucose-grown cells) were assigned into three GO categories: biological process, cellular component and molecular function. In total, 66 GO terms were found to be enriched in up-regulated DEGs [expression analysis systematic explorer (EASE) score < 0.1], of which 31 were involved with biological process, 21 with molecular function and 14 with cellular compartment (Additional file 1: Table S3). Besides, among the 46 GO terms enriched in down-regulated DEGs (EASE score < 0.1), 23 were involved with biological process, 13 with molecular function and 10 with cellular compartment (Additional file 1: Table S4).

Additionally, 10 and 30 KEGG pathways were found associated with the up-regulated and down-regulated DEGs, respectively. Notably, the genes up-regulated in acetate were involved in pathways associated with oxidative phosphorylation, mitogen-activated protein kinase (MAPK) signalling pathway, tricarboxylic acid (TCA) cycle, nucleotide excision repair, peroxisome, homologous recombination, endocytosis, protein processing in endoplasmic reticulum, carbon metabolism, glyoxylate and dicarboxylate metabolism (Table 2). Meanwhile, the down-regulated genes in acetate were primarily involved in pathways associated with biosynthesis of amino acids, biosynthesis of secondary metabolites, biosynthesis of antibiotics, as well as carbon metabolism and glycolysis (Table 2).

**Table 2:** List of enriched Kyoto encyclopedia of genes and genomes (KEGG) pathways in the differentially expressed genes (DEG)s of acetate-grown *C. glabrata* cells

KEGG entry	Pathway	Count	Frequency (%)	EASE score
Up-regulated DEGs				
cgr00190	Oxidative phosphorylation	25	3.36	5.76E−09
cgr04011	MAPK signalling pathway—yeast	13	1.75	2.54E−04
cgr00020	Citrate cycle (TCA cycle)	9	1.21	0.006
cgr03420	Nucleotide excision repair	10	1.34	0.0073
cgr04146	Peroxisome	10	1.34	0.0073
cgr03440	Homologous recombination	7	0.94	0.011
cgr04144	Endocytosis	14	1.88	0.0133
cgr04141	Protein processing in endoplasmic reticulum	14	1.88	0.047
cgr00630	Glyoxylate and dicarboxylate metabolism	6	0.81	0.0519
cgr01200	Carbon metabolism	16	2.15	0.0596
Down-regulated DEGs				
cgr01230	Biosynthesis of amino acids	69	9.43	8.67E−29
cgr01100	Metabolic pathways	180	24.59	2.96E−28
cgr01110	Biosynthesis of secondary metabolites	100	13.66	5.22E−21
cgr01130	Biosynthesis of antibiotics	70	9.56	6.25E−13
cgr01210	2-Oxocarboxylic acid metabolism	23	3.14	2.32E−10
cgr00260	Glycine, serine and threonine metabolism	18	2.46	1.28E−07
cgr00300	Lysine biosynthesis	11	1.5	4.06E−07
cgr00290	Valine, leucine and isoleucine biosynthesis	10	1.37	7.62E−06
cgr00400	Phenylalanine, tyrosine and tryptophan biosynthesis	12	1.64	1.41E−05
cgr00270	Cysteine and methionine metabolism	16	2.19	4.55E−05
cgr00920	Sulfur metabolism	8	1.09	4.20E−04
cgr00450	Selenocompound metabolism	7	0.96	0.0017
cgr00100	Steroid biosynthesis	9	1.23	0.0023
cgr00670	One carbon pool by folate	8	1.09	0.0071
cgr00510	N-Glycan biosynthesis	11	1.5	0.0099
cgr00514	Other types of *O*-glycan biosynthesis	6	0.82	0.012
cgr00564	Glycerophospholipid metabolism	12	1.64	0.0184
cgr00680	Methane metabolism	9	1.23	0.0195
cgr00010	Glycolysis/gluconeogenesis	14	1.91	0.0251
cgr00340	Histidine metabolism	6	0.82	0.0287
cgr00770	Pantothenate and CoA biosynthesis	7	0.96	0.0289
cgr00220	Arginine biosynthesis	7	0.96	0.0289
cgr00460	Cyanoamino acid metabolism	4	0.55	0.0303
cgr00230	Purine metabolism	21	2.87	0.0405
cgr01200	Carbon metabolism	23	3.14	0.0468
cgr00860	Porphyrin and chlorophyll metabolism	7	0.96	0.0508
cgr00261	Monobactam biosynthesis	3	0.41	0.0665
cgr00660	C5-Branched dibasic acid metabolism	3	0.41	0.0665
cgr00051	Fructose and mannose metabolism	7	0.96	0.0808
cgr00240	Pyrimidine metabolism	16	2.19	0.0929

### Differential expressed genes associated with carbon metabolism in acetate-grown *C. glabrata *cells

In the present study, the expression of high numbers of carbon metabolism genes were altered in acetate-grown *C. glabrata*. In the presence of acetate, glycolytic genes such as *PFK1*, *PFK2*, *TDH3*, *ENO1*, 3-phosphoglycerate kinase (*PGK1*), and pyruvate kinase (*CDC19*) were all down-regulated, ranging from 2.03-fold to 3.15-fold (Table 3). Although most of the reactions and enzymes are shared between glycolysis and the gluconeogenesis, two enzyme genes exclusive to gluconeogenesis (*FBP1*, *PCK1*) were significantly up-regulated 7.35-fold and 5.64-fold, respectively (Table 3). In addition, metabolic enzyme genes from the glyoxylate cycle were up-regulated in acetate-grown *C. glabrata* cells (Table 3). These include *ICL1* (4.58-fold), *MLS1* (2.56-fold), *CIT1* (2.11-fold) and putative aconitate hydratase (*ACO1*) (1.63-fold). Also, genes encoding TCA cycle enzymes such as isocitrate dehydrogenase (*IDP2*), α-ketoglutarate dehydrogenase complex (*KGD1*, *KGD2*), succinate dehydrogenases (*SDH1*, *SDH2* and *SHD4*) were significantly up-regulated (Table 3).

**Table 3:** Statistically significant differential gene expressions associated with carbon metabolism in acetate-grown *C. glabrata* (q value < 0.05)

Systematic name	Genes	Description	Fold change (log2)
Up-regulated genes associated with carbon metabolism in acetate-grown *C. glabrata*			
CAGL0H04939G	*FBP1*	Fructose 1,6-bisphosphatase	7.35
CAGL0H06633G	*PCK1*	Phosphoenolpyruvate carboxykinase	5.64
CAGL0J03058G	*ICL1*	Isocitrate lyase	4.58
CAGL0C03223G	*SDH2*	Succinate dehydrogenase	4.16
CAGL0M06963G	*SOL2*	Suppressor of Los1-1	2.68
CAGL0L03982G	*MLS1*	Malate synthase	2.56
CAGL0G08712G	*KGD1*	α-Ketoglutarate dehydrogenase complex	2.51
CAGL0H03993G	*CIT1*	Citrate synthase	2.11
CAGL0F05863G	*SDH4*	Succinate dehydrogenase	1.98
CAGL0J00847G	*SDH1*	Succinate dehydrogenase	1.92
CAGL0L03740G	*RKI1*	Ribose-5-phosphate isomerase	1.92
CAGL0B04917G	*IDP2*	Isocitrate dehydrogenase	1.89
CAGL0E01287G	*KGD2*	α-Ketoglutarate dehydrogenase complex	1.73
CAGL0D06424G	*ACO1*	Putative aconitate hydratase	1.63
CAGL0K10868G	*CTA1*	Putative catalase A	1.60
Down-regulated genes associated with carbon metabolism in acetate-grown *C. glabrata*			
CAGL0K00825G	*SER2*	Phosphoserine phosphatase	− 3.52
CAGL0I09284G	*SHM1*	Glycine hydroxymethyltransferase	− 3.42
CAGL0K06787G	*PYC2*	Pyruvate carboxylase	− 3.37
CAGL0L11088G	*YOR283W*	Phosphatase	− 3.25
CAGL0M12034G	*CDC19*	Pyruvate kinase	− 3.18
CAGL0J00451G	*TDH3*	Glyceraldehyde-3-phosphate dehydrogenase	− 3.15
CAGL0K08580G	*AAT1*	Aspartate aminotransferase	− 3.11
CAGL0F08041G	*PFK1*	Phosphofructokinase	− 3.08
CAGL0H05137G	*ALD5*	Aldehyde dehydrogenase	− 2.90
CAGL0L10758G	*PFK2*	6-Phosphofructokinase	− 2.73
CAGL0I05126G	*ILV1*	l-Threonine ammonia-lyase	− 2.56
CAGL0I05500G	*PRS2*	Ribose phosphate diphosphokinase	− 2.28
CAGL0C05181G	*PRS5*	Ribose phosphate diphosphokinase	− 2.29
CAGL0E06358G	*GPM1*	Phosphoglycerate mutase	− 2.24
CAGL0I02486G	*ENO1*	Enolase	− 2.09
CAGL0H08327G	*TPI1*	Triose-phosphate isomerase	− 2.07
CAGL0D06402G	*MET15*	*O*-Acetyl homoserine sulfhydrylase	− 2.05
CAGL0L07722G	*PGK1*	3-Phosphoglycerate kinase	− 2.03
CAGL0L03311G	*SHB17*	Sedoheptulose-bisphosphatase	− 2.01
CAGL0D04356G	*GCV1*	Glycine dehydrogenase	− 1.96
CAGL0F01749G	*SHM2*	Serine hydroxymethyltransferase	− 1.94
CAGL0H07579G	*HXK2*	Hexokinase	− 1.84
CAGL0J01441G	*ADH2*	Alcohol dehydrogenase	− 1.55
CAGL0J09504G	*MET12*	Methylenetetrahydrofolate reductase	− 1.51

Data of differential gene expressions (q value < 0.05) was obtained from RNA-sequencing analysis. Fold change (log2) was calculated based on the gene expression of acetate-grown *C. glabrata* to glucose-grown *C. glabrata* (control)

### Proteomic landscape of acetate-grown *C. glabrata*

**Fig. 2:**
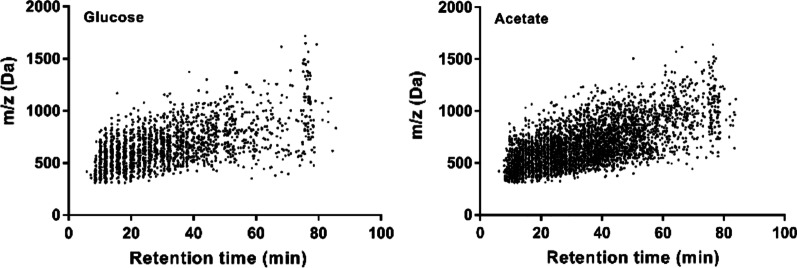
Scatter plot indicates the mass versus retention time of the peptides identified from glucose-grown and acetate-grown *C. glabrata*. Most of the separated peptides from these two growth conditions had masses between 300 and 1500 Da, and most of the separated peptides eluting from 10 to 80 min

**Fig. 3:**
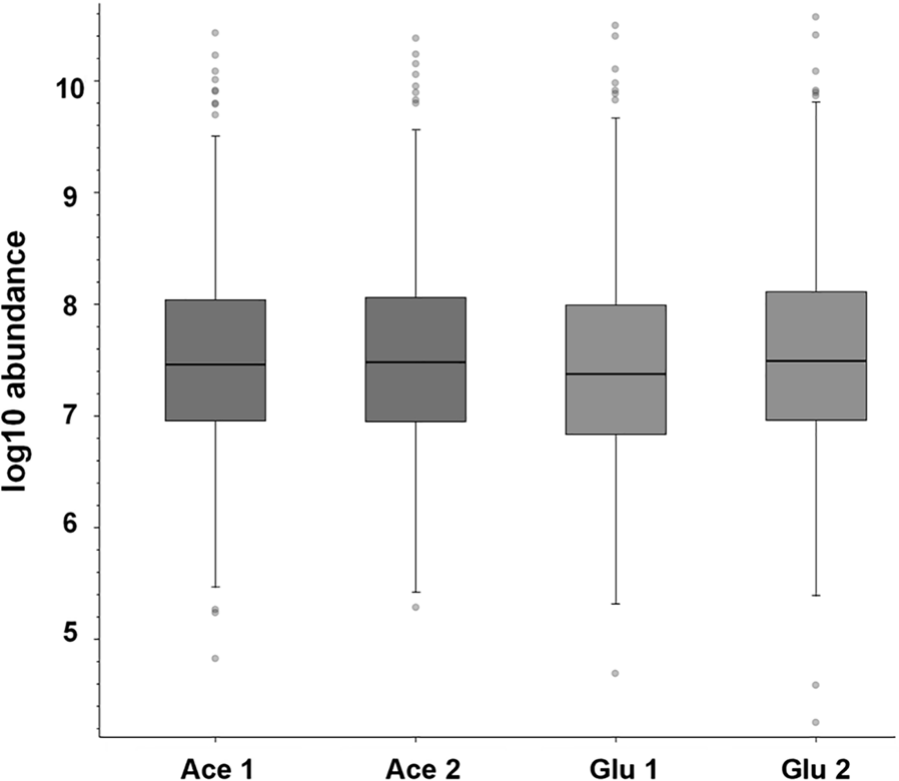
Abundance and detection range of the proteins identified from glucose-grown and acetate-grown *C. glabrata*. Highly abundance proteins identified were up to 5-log higher abundance in comparison to the lowest abundance proteins identified

To investigate the impact of alternative carbon source on the proteome of *C. glabrata*, cells were exposed to 2% (w/v) glucose or 2% (w/v) acetate for 24 h and analyzed with label-free quantitative proteomics. A total of three technical replicates for each of the two biological replicates of the growth conditions were analyzed. As shown in Fig. 2, majority of the separated peptides had masses between 300 and 1500 Da, with most of the peptides eluting from 10 to 80 min. On the other hand, highly abundance proteins (e.g. glyceraldehyde-3-phosphate dehydrogenase, enolase and phosphoglycerate kinase) identified were up to 5-log higher abundance in comparison to the lowest abundance proteins (e.g. proteasome subunit beta type 2 and putative ubiquitin-conjugating enzyme), indicating a good and acceptable protein detection range in both growth conditions (Fig. 3).

**Fig. 4:**
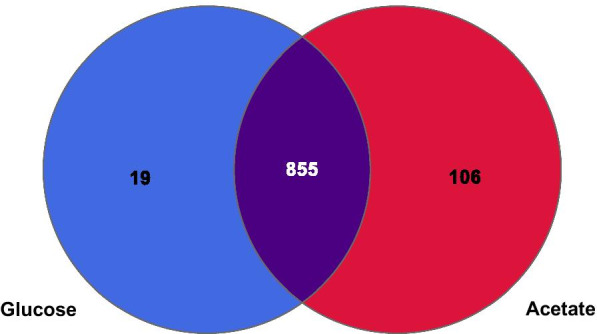
Venn diagram showing total proteins identified between the two treatment groups, glucose-grown and acetate-grown *C. glabrata*. A total of 855 proteins identified were expressed in both growth conditions, while only 19 and 106 proteins identified were expressed exclusively in glucose-grown and acetate-grown *C. glabrata* cells, respectively

**Fig. 5:**
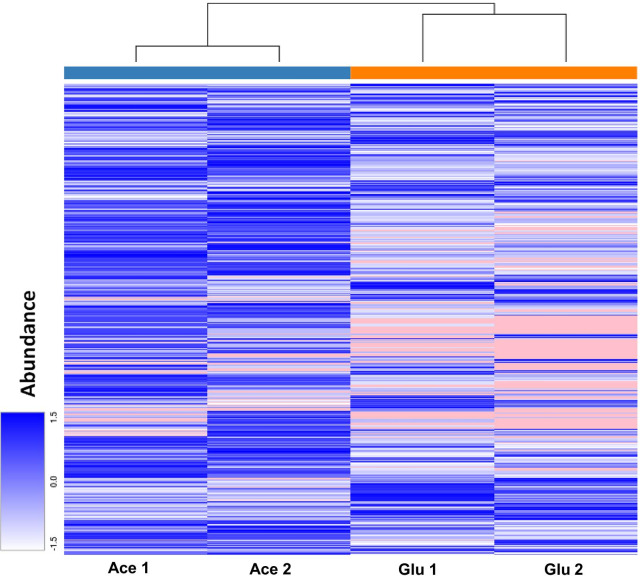
Hierarchical clustering analysis of the proteins identified from glucose-grown and acetate-grown *C. glabrata* cells. Hierarchical clustering analysis demonstrated a clear divergent in the proteomes of the glucose-grown and acetate-grown *C. glabrata* cells, with only minimal differences between the biological replicates

In the present study, label-free quantitative proteomic analysis uncovered 980 proteins, with only a limited amount of proteins identified “exclusively” under glucose- or acetate-grown conditions (note that “exclusive” expression meant that the level of a given protein was below the detection limit for this analysis under one condition, rather than not being expressed under that condition). A total of 106 proteins were expressed exclusively in acetate-grown *C. glabrata* cells, while only 19 proteins were exclusive to the glucose-grown cells (Fig. 4). Hierarchical clustering analysis demonstrated a clear difference in the proteomes of the glucose-grown and acetate-grown *C. glabrata* cells, with only minimal variance between the biological replicates (Fig. 5). Although the majority of identified proteins were not significantly regulated, a small portion of identified proteins (192 significantly up-regulated, 50 significantly down-regulated in acetate) was differentially expressed between the two growth conditions (log2 fold change > 1.5 and q value < 0.05).

### Functional enrichment of the DEPs

Functional enrichment analysis was also performed on the DEPs to detect the significantly enriched GO and KEGG pathways using DAVID Bioinformatics Resource 6.8 tool. A total of 22 and two GO terms were strongly enriched in the up- and down-regulated proteins in acetate, respectively, with EASE Score less than 0.1 (Additional file 1: Table S5). Pathway analysis revealed that 14 and four KEGG pathways were also significantly enriched (Table 4). Proteins associated with metabolic pathways were the most represented in acetate-grown *C. glabrata*. This included pathways involved in oxidative phosphorylation, carbon metabolism, citrate cycle, pyruvate metabolism, 2-oxocarboxylic acid metabolism, gluconeogenesis, glyoxylate and dicarboxylate metabolism. Interestingly, KEGG pathways associated with biosynthesis of secondary metabolites, antibiotics and amino acids were detected as up-regulated at the protein level only (Table 4).

**Table 4:** List of enriched Kyoto encyclopedia of genes and genomes (KEGG) pathways in the differentially expressed proteins (DEPs) of acetate-grown *C. glabrata*

Pathway	Count	Frequency (%)	EASE score
Up-regulated			
Metabolic pathways	60	35.7	1.50E−12
Biosynthesis of secondary metabolites	34	20.2	1.00E−08
Carbon metabolism	19	11.3	1.50E−07
Citrate cycle (TCA cycle)	11	6.5	2.40E−07
Biosynthesis of antibiotics	26	15.5	6.50E−07
Oxidative phosphorylation	14	8.3	5.30E−06
Pyruvate metabolism	9	5.4	9.60E−05
Biosynthesis of amino acids	16	9.5	1.30E−04
2-Oxocarboxylic acid metabolism	8	4.8	7.80E−04
Glyoxylate and dicarboxylate metabolism	6	3.6	2.00E−03
Glycolysis/gluconeogenesis	7	4.2	1.80E−02
Pentose and glucuronate interconversions	3	1.8	2.80E−02
Arginine biosynthesis	4	2.4	3.40E−02
Alanine, aspartate and glutamate metabolism	4	2.4	9.40E−02
Down-regulated			
Metabolic pathways	14	29.8	6.00E−03
Biosynthesis of antibiotics	7	14.9	1.90E−02
Cysteine and methionine metabolism	3	6.4	5.30E−02
Biosynthesis of secondary metabolites	7	14.9	7.20E−02

### Differential expressed proteins associated with carbon metabolism in acetate-grown *C. glabrata *cells

A large portion of the proteomic response in acetate-grown *C. glabrata* involved the increase abundance of proteins associated with carbon metabolism, particularly in gluconeogenesis and the glyoxylate cycle (Table 5). Notably, glyoxylate cycle enzymes, which include isocitrate lyase (6.64-fold), malate synthase (2.59-fold), malate dehydrogenase (1.53-fold), citrate synthase (3.30-fold) and aconitase hydratase (6.64-fold), succinate dehydrogenase (2.62-fold) and fumarate hydratase (1.60-fold), were all up-regulated. Similarly, the gluconeogenic enzymes fructose 1,6-bisphosphatase and phosphoenolpyruvate carboxykinase were up-regulated 3.20-fold and 4.20-fold, respectively. This increase in gluconeogenesis and the glyoxylate cycle reflects the involvement of these pathways in the production of hexoses and pentoses from acetate (e.g. for cell wall biosynthesis and nucleotide biosynthesis, respectively). Also, acetyl-CoA synthetase, an important enzyme involved in acetate metabolism was also up-regulated (1.54-fold) in response to acetate. In addition, proteins potentially associated with the production of anaplerotic precursors for glucose, such as the pyruvate dehydrogenase complex, alcohol dehydrogenase and alanine-glyoxylate transaminase, were also induced.

**Table 5:** Statistically significant differential protein expressions associated with carbon metabolism in acetate-grown *C. glabrata* (q value < 0.05)

Systematic name	Proteins	Description	Fold change (log2)
CAGL0J03058g	Icl1	Isocitrate lyase	6.64
CAGL0D06424g	Aco1	Aconitate hydratase	6.64
CAGL0K12518g	Agx1	Alanine-glyoxylate transaminase	6.64
CAGL0L05478g	Rpe1	Ribulose-phosphate 3-epimerase Transaldolase	6.64
CAGL0L12254g	Alt1	l-Alanine:2-oxoglutarate aminotransferase	6.64
CAGL0H06853g	Adh6	NADP-dependent alcohol dehydrogenase VI	6.64
CAGL0H06633g	Pck1	Phosphoenolpyruvate carboxykinase	4.20
CAGL0B03663g	Cit1	Citrate synthase	3.30
CAGL0H04939g	Fbp1	Fructose 1,6-bisphosphatase	3.20
CAGL0C03223g	Sdh2	Succinate dehydrogenase	2.62
CAGL0L03982g	Mls1	Malate synthase	2.59
CAGL0J10186g	Lat1	Pyruvate dehydrogenase complex	2.33
CAGL0B04917g	Idp2	Isocitrate dehydrogenase	2.30
CAGL0L06842g	Thi3	RNA polymerase II activating transcription factor	1.91
CAGL0K04235g	Tal1	Transaldolase	1.87
CAGL0L12078g	Pda1	Pyruvate dehydrogenase (acetyl-transferring)	1.73
CAGL0I07139g	Lsc1	Succinate-CoA ligase (ADP-forming)	1.65
CAGL0A01045g	Fum1	Fumarate hydratase	1.60
CAGL0L00649g	Acs1	Acetyl-coenzyme A synthetase	1.54
CAGL0L05236g	Mdh1	Malate dehydrogenase	1.53
CAGL0G02673g	Idh1	Isocitrate dehydrogenase	1.50

Data of differential protein expressions (q value < 0.05) was obtained from proteomic analysis. Fold change (log2) was calculated based on the protein expression of acetate-grown *C. glabrata* to glucose-grown *C. glabrata* (control)

## Discussion

The main goal of this study is to decipher a global view of the metabolic response of the major fungal pathogen *C. glabrata*, in the presence of acetate as the sole carbon source, through a combination of high-throughput transcriptomic and proteomic profiling. Historically, *C. glabrata* belongs to the Saccharomyces clade and is phylogenetically more closely related to *S. cerevisiae* than other *Candida* species [35]. However, further examination reveals that *C. glabrata* does not act exactly like the baker’s yeast. For instance, the capability of carbon assimilation has been reduced significantly in comparison to *S. cerevisiae*. In fact, *C. glabrata* is unable to utilize many carbon sources such as galactose, maltose, lactose, sucrose and raffinose and mainly rely on glucose and trehalose [36], it has been shown to be able to utilize certain alternative carbon sources such as carboxylic acids, fatty acids and alcohols [13]. Furthermore, adaptation to these alternative carbon sources also induced physiological changes associated with pathogenicity of *C. glabrata*. Although *C. glabrata* is not commonly used in biotechnology field, it is an important microorganism used for cost-effective industrial production of pyruvate, from carbon sources like glucose, acetate and oxaloacetate [37–39].

**Fig. 6:**
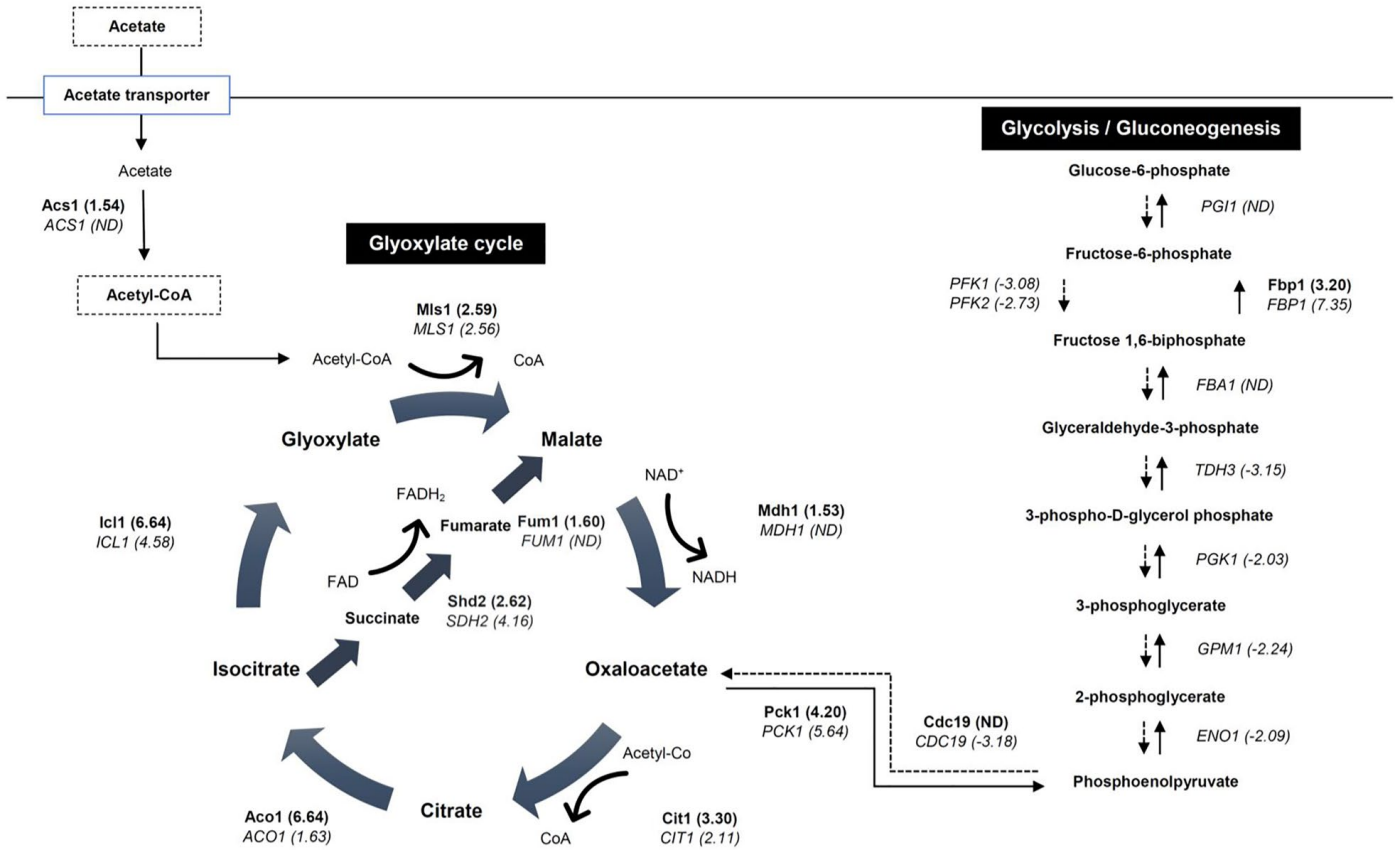
Induction of the glyoxylate cycle and gluconeogenesis in acetate-grown *C. glabrata*. The glyoxylate cycle and glycolysis (dashed arrows)/gluconeogenesis (full line arrows) are shown, along with the log2 fold changes of transcripts (italic) and proteins (bold) obtained from the transcriptomic and proteomic analyses in this study, respectively. ND indicates the transcript or protein is not detected

In human, acetate is present in the blood at the range of 0.1–0.5 mM [40] and has been shown to be relevant within macrophages and neutrophils [5, 15]. Previously, it has been shown that *C. glabrata* is able to utilize acetate [9, 12, 41]. In addition, putative acetate permease gene *ADY2* was highly induced in macrophages-engulfed *C. glabrata*, signifying the importance of acetate in the survival of engulfed-*C. glabrata*. Here, it was demonstrated that *C. glabrata* attunes the alternative carbon metabolism in response to acetate through upregulation and downregulation of key metabolic pathways at both the protein and transcript levels (Fig. 6).

The data suggest that *C. glabrata* regulates its carbon metabolism in favor of production of hexoses during glucose starvation. The majority of the induced pathways in acetate-grown *C. glabrata* were associated with alternative carbon metabolism, namely gluconeogenesis and the glyoxylate cycle. In fact, *C. glabrata* appears to employ a pattern of carbon adaptation that resembles other fungal pathogens such as *C. albicans*, *Candida orthopsilosis* (a subspecies of *C. parapsilosis* complex), *C. neoformans* and *Paracoccidioides brasiliensis* during glucose starvation [4, 19, 42]. Phagocytosed *C. albicans* and *C. neoformans* cells appear to encounter glucose deprivation following phagocytosis by the immune cells, and they counteract this stress by replenishing the glucose intermediates from alternative carbon sources, probably through upregulation of genes from the three interconnected pathways: β-oxidation of fatty acids, the glyoxylate cycle and gluconeogenesis [4,42] Also, carbon-starved *P. brasiliensis* induces a metabolic reprogramming that mirrors *C. albicans* and *C. neoformans*, resulted in drastic changes in the alternative carbon metabolism pathways [19].

The glyoxylate cycle is basically a shunt in the TCA cycle, with which it shares many of its metabolic enzymes [45]. The glyoxylate cycle represented one of the most upregulated pathways in *C. glabrata* during the change of carbon source from glucose to acetate. In general, all transcripts and proteins from the glyoxylate cycle like isocitrate lyase (*ICL1*), malate synthase (*MLS1*), aconitase (*ACO1*) and citrate synthase (*CIT1*) were highly induced at the transcript level (1.63- to 4.58-fold) and protein level (2.69- to 6.64-fold), respectively. In addition, fumarate hydratase was also induced in acetate-grown *C. glabrata*. Glyoxylate cycle bypasses the two decarboxylation steps in TCA cycle and allows utilization of two-carbon compounds acetyl-CoA, which is converted from the assimilated acetate by acetyl-CoA synthetase [46]. In this way, flux through the glyoxylate cycle promotes carbon assimilation during the utilization of alternative carbon sources, whilst flux through the TCA cycle maximizes energy generation. It is worth noting that acetyl-CoA synthetase was also up-regulated in acetate-grown *C. glabrata* cells. Ultimately, the net oxaloacetate produced from the two-carbon compounds (e.g., acetate) can serve as an intermediate to replenish the TCA cycle in the absence of glucose, or fuel gluconeogenesis for production of glucose intermediate and cellular building blocks [7, 47]. Increased expression of the glyoxylate cycle has been reported in macrophage-engulfed *C. glabrata* [5]. However, this observation could be merely attributed by the general stresses imposed on the fungal cells by the phagocytes. Results obtained strongly suggested that *C. glabrata* could potentially utilize acetate within macrophages, and the presence of acetate clearly induced the glyoxylate cycle and gluconeogenesis.

Generally, *C. glabrata* employs a carbon flow via classical metabolic pathways such as gluconeogenesis, the glyoxylate cycle and TCA cycle following acetate assimilation. Moreover, suppression of glycolytic growth is a hallmark sign of glucose deprivation in *C. glabrata* [5]. Other metabolic pathways that were most affected also include nucleotide and amino acid metabolism. In this study, biosynthesis of amino acids including serine (*SER2*), glycine (*GCV1*), methionine (*MET12*, *MET15*) and isoleucine (*ILV1*) were all down-regulated, potentially to conserve energy amidst glucose starvation. In addition, l-alanine:2-oxoglutarate aminotransferase was induced in acetate-grown *C. glabrata*, thus facilitates the conversion of alanine to pyruvate, an intermediate for the glyoxylate shunt. Breakdown of these glucogenic amino acids are likely are being used to produce precursors needed for alternative carbon metabolism in many fungal pathogens [48]. Genes associated with pentose phosphate pathway such as *PRS2*, *PRS5* and *SHB17* and proteins such as ribulose-phosphate 3-epimerase and transaldolase were also down-regulated in response to acetate utilization, thus limiting the nucleotide biosynthetic process in *C. glabrata*.

Oxidative phosphorylation was also induced in acetate-grown *C. glabrata* at protein and transcripts levels. The process of oxidative phosphorylation takes place in the mitochondria and is responsible for generation of the primary energy source, adenosine triphosphate (ATP) [49]. In this study, acetate utilization via the glyoxylate cycle possibly replenishes the TCA cycle intermediate (i.e., oxaloacetate) and also produces cofactors such as nicotinamide adenine dinucleotide (NADH) and flavin adenine dinucleotide (FADH_2_). Up-regulation of oxidative phosphorylation in acetate-grown *C. glabrata* suggested that this fungal pathogen could generate ATP required for its survival and the anabolic metabolism from NADH and FADH_2_ via oxidative phosphorylation in the electron transport chain system.

Production of glucose from glucose intermediates also requires a functional gluconeogenesis. In this study, two metabolic enzymes exclusive to gluconeogenesis, fructose 1,6-biphosphatase (*FBP1*) and phosphoenolpyruvate carboxykinase (*PCK1*) were induced 7.35- and 5.64-fold at the transcript level, as well as 3.20- and 4.20-fold at the protein level, respectively (Tables 3, 5). Both of these enzymes contribute to the conversion of oxaloacetate from the glyoxylate cycle into hexose phosphates via gluconeogenesis. Catabolism and anabolism of glucose in fungi are controlled by glycolysis and gluconeogenesis, respectively [50]. In the absence of glucose, catabolism of glucose via glycolysis appears to be repressed, as demonstrated by the down-regulation of transcripts of many glycolytic enzymes such as phosphofructokinase (*PFK1* and *PFK2*), hexokinase (*HXK2*), and pyruvate kinase (*CDC19*). However, these glycolytic enzymes were not detected at the protein level in this study.

Other than the glyoxylate cycle and gluconeogenesis enzymes, the transcript of the TCA cycle enzymes such as isocitrate dehydrogenase (*IDP2*), succinate dehydrogenase (*SDH2*) and α-ketoglutarate dehydrogenase complex (*KGD1*, *KGD2*) were up-regulated in the absence of glucose. It has been reported that a subset of *C. albicans* within the cell populations activated the glyoxylate cycle and gluconeogenic growth during early phagocytosis by the immune cells. However, they also expressed glycolytic mechanisms that are required for the systemic disease progression [43]. The same reasoning might also be applicable to acetate-grown *C. glabrata*. The fungal pathogen could potentially express TCA cycle enzymes concurrently with gluconeogenic enzymes in preparation for the colonization of the host, once glucose availability is no longer a limiting factor.

Mitogen-activated protein kinase pathways are conserved signalling cascades in eukaryotes that are involved in the transduction of extracellular or environmental stimuli for transcriptional regulation [51, 52]. In fungi, MAPK signalling pathways have been implicated to be involved in a wide range of processes such as fungal pathogenicity, mating, morphogenesis, stress resistance and cell wall integrity [53–55]. In this study, MAPK signalling components were also up-regulated in acetate-grown *C. glabrata* (*STE11*, *STE12*, *STE20*, *SLN1*, *YPD1*, *SSK1*, *SLT2*, *SWI4*, *MID2*, *MSN2*, *MSG5*, *BEM1* and *MCM1*) compared to glucose-grown cells (Table 2). The *STE* vegetative pathway has been reported to play an important role in the maintenance of cell wall integrity of *S. cerevisiae* via Ste11, Ste12 (with the DNA binding protein Mcm1) and Ste20, and these transcription factors are responsible for the transcription of genes related to the conservation of cell wall integrity [56–58]. In *C. glabrata*, although both Ste11 and Ste12 are largely expendable for the cell wall integrity (unlike in *S. cerevisiae*), Ste20 is crucial in maintaining the cell wall integrity of *C. glabrata* [59–61]. Similarly, increased *C. glabrata SLT2* expression has been linked to the elevated chitin level and echinocandin tolerance in *C. glabrata* [62], signifying the importance of *SLT2* and its downstream transcriptional regulator (e.g. Swi4) in the rescue of cell wall integrity and tolerance to cell-damaging stresses such as Congo red, Calcofluor white, caspofungin and micafungin [62–64].

The orthologous gene *MID2* was also induced in acetate-grown cells. This implies that *C. glabrata* could activate *PKC1*-*MPK1* signal transduction pathway via *MID2* similar to *S. cerevisiae* for maintenance of cell wall integrity [65]. The high-osmolarity glycerol (HOG) pathway, which is normally inactivated by three phospho-relay components Sln-Ypd1-Ssk1 under normal condition mediates cellular response to osmotic shocks [66]. It is worth mentioning that *SLN1*, *YPD1* and *SSK1* were induced in acetate-grown *C. glabrata*, suggesting that HOG pathway is likely inactivated in this condition. Taken together, results obtained highly suggest that the fungal cells may employ MAPK signalling to control its transcriptional regulation in order to modulate fitness attributes of *C. glabrata* in response to acetate, as demonstrated in our study [13], particularly on cell wall structure, stress and antifungal resistance. Regulation of fitness and pathogenic attributes via MAPK pathways could potentially assist in *C. glabrata* ability to resist phagocytic killing [67–69].

In addition, ethanol catabolism (*ADH2*, *ALD5*) were also inhibited in the presence of acetate as the sole carbon source. Alcohol dehydrogenase (*ADH2*) catalyses the breakdown of ethanol into acetaldehyde, which could be converted to acetate by aldehyde dehydrogenase (*ALD5*) [46, 70]. It is worth to highlight that minor discrepancies between transcript and protein abundances were observed in pathways associated with biosynthesis of secondary metabolites and amino acids. Biosynthesis of amino acids such as valine, leucine, isoleucine, phenylalanine, tyrosine, tryptophan, cysteine, methionine, histidine and arginine were all repressed at the transcript levels, whilst protein abundance levels were increased. Differences between transcriptome and proteome data could be attributed, at least in part to the translational regulation. In fact, there were precedents for mRNAs associated with amino acid biosynthesis such as *CPA1* and *GCN4* to be translationally regulated in yeast cells [71, 72].

## Conclusion

Overall, RNA-sequencing and label-free quantitative proteomic analyses had successfully revealed the first transcriptomic and proteomic responses of *C. glabrata* in the presence of acetate as the sole carbon source. Here, we showed that *C. glabrata* mainly depended on the glyoxylate cycle and gluconeogenic growth to replenish glucose intermediates and generate energy from acetate. This results also strengthen the idea that *C. glabrata* could potentially survive within macrophages, owing to its ability to switch into alternative carbon metabolism. In addition, investigation of the reprogramming of carbon metabolism in *C. glabrata* is imperative to understand the metabolic regulation and pathogenesis of this fungal pathogen in glucose deprivation condition. It is worth mentioning that only a single strain of *C. glabrata* and carbon source were analyzed and this is a limitation of the study. Therefore, potential future study focuses on clinical strains of *C. glabrata* and other physiologically relevant carbon sources are certainly warranted.

## Supplementary Information

**Additional file 1: Table S1.** List of primers used in qPCR experiments. **Table S2.** Coefficient of determination (r^2^) between biological replicates for glucose- and acetate-grown *C. glabrata*. **Table S3.** List of enriched GO terms associated with up-regulated DEGs of acetate-grown *C. glabrata* cells. **Table S4.** List of enriched GO terms associated with down-regulated DEGs of acetate-grown *C. glabrata* cells. **Table S5.** List of enriched GO terms associated with DEPs of acetate-grown *C. glabrata*.

## Data Availability

The datasets supporting the conclusions of this article are available in the (1) European Nucleotide Archive (RNA-sequencing data, study accession number: PRJEB33880) and (2) ProteomeXchange Consortium via the PRIDE partner repository (proteomics data, dataset identifier: PXD014916 and 10.6019/PXD014916).

## Abbreviations

AGC: Automatic gain control
ATCC: American Type Culture Collection
ATP: Adenosine triphosphate
CHAPS: 3-[(3-Cholamidopropyl)dimethylammonio]-1-propanesulfonate
CT: Cycle threshold
DAVID: Database for Annotation, Visualization and Integrated Discovery
DEGs: Differentially expressed genes
DEPs: Differentially expressed proteins
DTT: Dithiothreitol, ENA: European Nucleotide Achieve
FADH_2_: Flavin adenine dinucleotide (hydroquinone form)
FDR: False discovery rate
FPKM: Fragments per kilobase of transcript per million mapped fragments
GO: Gene ontology
HCD: High-energy collisional dissociation
HOG: High-osmolarity glycerol
iTRAQ: Isobaric tagging for relative and absolute quantification
KEGG: Kyoto Encyclopedia of Genes and Genomes
MAPK: Mitogen-activated protein kinase
NADH: Nicotinamide adenine dinucleotide (reduced form)
NCAC: Non-*Candida albicans Candida*
NCBI: National Center for Biotechnology Information
NGS: Next generation sequencing
PBS: Phosphate-buffered saline
SC: Synthetic complete
SPE: Solid phase extraction
TCA: Tricarboxylic acid
TCEP: Tris(2-carboxyethyl) phosphine
UHPLC: Ultra-high-performance liquid chromatography
YNB: Yeast nitrogen base
YPD: Yeast extract peptone dextrose
