# Supplementary material for: Transcriptomic and proteomic profiling revealed reprogramming of carbon metabolism in acetate-grown human pathogen Candida glabrata
Source: J Biomed Sci. 2021 Jan 2;28:1. doi: 10.1186/s12929-020-00700-8 (PMC7778802; doi:10.1186/s12929-020-00700-8)
Supplement: Supplementary file 1 — Additional file 1: Table S1. List of primers used in qPCR experiments. Table S2. Coefficient of determination (r2) between biological replicates for glucose- and acetate-grown C. glabrata. Table S3. List of enriched GO terms associated with up-regulated DEGs of acetate-grown C. glabrata cells. Table S4. List of enriched GO terms associated with down-regulated DEGs of acetate-grown C. glabrata cells. Table S5. List of enriched GO terms associated with DEPs of acetate-grown C. glabrata. [file 12929_2020_700_MOESM1_ESM.docx]

**S1 Table:** **List of primers used in qPCR experiments.**

| Genes | Systematic names | Primers | Primer sequences (5’ – 3’) | References |
| --- | --- | --- | --- | --- |
| *ICL1* | CAGL0J03058g | ICL1-F  ICL1-R | CCTCCGTCTCCAACAAGCAA  GGCAAGTCCCAGTCGAAGAA | This study |
| *MLS1* | CAGL0L03982g | MLS1-F  MLS1-R | ATGGAGCATCACTTGGAGGC  GGAAAGCAGCAGGCAAAGTC | This study |
| *PCK1* | CAGL0H06633g | PCK1-F  PCK1-R | TGTCCACCACAACGTGCTAA  TCGTCGTCACCGATCAACAA | This study |
| *FBP1* | CAGL0H04939g | FBP1-F  FBP1-R | GGTCGCAGATGTCCACAGAA  AACTGCTTTACCACCGGCTT | This study |
| *SNF3* | CAGL0J09020g | SNF3-F  SNF3-R | AGCAGCTGGCCATATTGGTT  ATGTTATGTGCCCCCACCTG | This study |
| *CIT1* | CAGL0H03993g | CIT1-F  CIT1-R | AGACCTGCTGGGTAAGTTGC  CCCAATAGGTGGGCCAAGTT | This study |
| *SIP4* | CAGL0L03377g | SIP4-F  SIP4-R | TGAGATTGGCCAGTTGGCAT  ATGTGCCGTGTCCAAGGTAG | This study |
| *GSM1* | CAGL0L03674g | GSM1-F  GSM1-R | AGGCCAGCTGTGTAGGGATA  GCTGACACATTGACTTCGGC | This study |
| *PFK1* | CAGL0F08041g | PFK1-F  PFK1-R | ATGCCAGGTCATGCTCAACA  CCAAAGCAGTGGCCTTGTTC | This study |
| *PFK2* | CAGL0L10758g | PFK2-F  PFK2-R | TACGAAGGTTTGGTCCGTGG  TTGGGCACCTAACAGACGAC | This study |
| *PFK27* | CAGL0E01529g | PFK27-F  PFK27-R | GCCATGAGTACGAACGACCA  CAACCCGACCAGGATGACAA | This study |
| *PYK1* | CAGL0M12034g | PYK1-F  PYK1-R | ATCACCAAGGTCATCTCCGC  GGGAGCAGATCTTACCAGCG | This study |
| *PYC2* | CAGL0K06787g | PYC2-F  PYC2-R | GAAACCAGCCGCTGCTAAAC  CAACATCAGCACCAGCAAGG | This study |
| *TDH3* | CAGL0G09383g | TDH3-F  TDH3-R | TCCAACGCTTCCTGTACCAC  CAGTCCTTGTGGGATGGACC | This study |
| *ENO1* | CAGL0I02486g | ENO1-F  ENO1-R | CTCTGACGCTTCCAAGTGGT  GCGGTCTTGAAGAAGTGGGA | This study |
| *ACT1* | CAGL0K12694g | ACT1-F  ACT1-R | ACCAACTGGGATGACATGGA  TCATTGGAGCCTCGGTCAAC | This study |

**S2 Table: Coefficient of determination (r^2^) between biological replicates for glucose- and acetate-grown *C. glabrata.***

| **Sample** | **Glucose-grown**  ***C. glabrata*** | | | **Sample** | **Acetate-grown**  ***C. glabrata*** | | |
| --- | --- | --- | --- | --- | --- | --- | --- |
|  | **Glu 1** | **Glu 2** | **Glu 3** |  | **Ace 1** | **Ace 2** | **Ace 3** |
| **Glu 1** |  | 0.9432 | 0.9495 | **Ace 1** |  | 0.9556 | 0.9531 |
| **Glu 2** | - |  | 0.9438 | **Ace 2** | - |  | 0.9477 |
| **Glu 3** | - | - |  | **Ace 3** | - | - |  |

**S3 Table:** **List of enriched GO terms associated with up-regulated DEGs of acetate-grown *C. glabrata* cells**

| **GO accession** | **GO terms** | **Count** | **Frequency (%)** | **EASE score** |
| --- | --- | --- | --- | --- |
| **Biological Process** | | | | |
| GO:0006122  GO:0009408  GO:0006511  GO:0042026  GO:0009060  GO:0016579  GO:0006123  GO:0006470  GO:0006397  GO:0006099  GO:0001927  GO:0006351  GO:0036003  GO:0007232  GO:0016567  GO:0031505  GO:0031087  GO:0000200  GO:0042176  GO:0006457  GO:0006301  GO:0000288  GO:0001402  GO:0034605  GO:0070816  GO:0032186  GO:0061429  GO:0051601  GO:0016197  GO:0061402  GO:0006458 | Mitochondrial electron transport, ubiquinol to cytochrome c  Response to heat  Ubiquitin-dependent protein catabolic process  Protein refolding  Aerobic respiration  Protein deubiquitination  Mitochondrial electron transport, cytochrome c to oxygen  Protein dephosphorylation  mRNA processing  Tricarboxylic acid cycle  Exocyst assembly  Transcription, DNA-templated  Positive regulation of transcription from RNA polymerase II promoter in response to stress  Osmosensory signalling pathway via Sho1 osmosensor  Protein ubiquitination  Fungal-type cell wall organization  Deadenylation-independent decapping of nuclear-transcribed mRNA  Inactivation of MAPK activity involved in cell wall organization or biogenesis  Regulation of protein catabolic process  Protein folding  Post-replication repair  Nuclear-transcribed mRNA catabolic process, deadenylation-dependent decay  Signal transduction involved in filamentous growth  Cellular response to heat  Phosphorylation of RNA polymerase II C-terminal domain  Cellular bud neck septin ring organization  Positive regulation of transcription from RNA polymerase II promoter by oleic acid  Exocyst localization  Endosomal transport  Positive regulation of transcription from RNA polymerase II promoter in response to acidic pH  De novo protein folding | 8  11  14  7  14  10  6  6  8  9  4  20  4  4  6  17  4  3  3  14  4  4  4  6  5  3  3  3  3  3  3 | 1.08  1.48  1.88  0.94  1.88  1.34  0.81  0.81  1.08  1.21  0.54  2.69  0.54  0.54  0.81  2.28  0.54  0.40  0.40  1.88  0.54  0.54  0.54  0.81  0.67  0.40  0.40  0.40  0.40  0.40  0.40 | 2.54E-05  3.50E-05  3.22E-04  8.52E-04  0.0011  0.0016  0.0019  0.0067  0.0073  0.0090  0.0093  0.0155  0.0209  0.0209  0.0334  0.0367  0.0376  0.0516  0.0516  0.0542  0.0593  0.0854  0.0854  0.0902  0.0918  0.0939  0.0939  0.0939  0.0939  0.0939  0.0939 |
| **Molecular Function** | | | | |
| GO:0043565  GO:0008121  GO:0004129  GO:0046872  GO:0004672  GO:0000981  GO:0003700  GO:0008270  GO:0020037  GO:0005546  GO:0001077  GO:0004843  GO:0003677  GO:0004865  GO:0004693  GO:0019237  GO:0000982  GO:0005524  GO:0008289  GO:0005034  GO:0008353 | Sequence-specific DNA binding  Ubiquinol-cytochrome-c reductase activity  Cytochrome-c oxidase activity  Metal ion binding  Protein kinase activity  RNA polymerase II transcription factor activity, sequence-specific DNA binding  Transcription factor activity, sequence-specific DNA binding  Zinc ion binding  Heme binding  Phosphatidylinositol-4,5-bisphosphate binding  Transcriptional activator activity, RNA polymerase II core promoter proximal region sequence-specific binding  Thiol-dependent ubiquitin-specific protease activity  DNA binding  Protein serine/threonine phosphatase inhibitor activity  Cyclin-dependent protein serine/threonine kinase activity  Centromeric DNA binding  Transcription factor activity, RNA polymerase II core promoter proximal region sequence-specific binding  ATP binding  Lipid binding  Osmosensor activity  RNA polymerase II carboxy-terminal domain kinase activity | 24  8  9  42  13  13  10  42  7  6  11  7  30  3  4  4  4  76  5  3  3 | 3.23  1.08  1.21  5.65  1.75  1.75  1.34  5.65  0.94  0.81  1.48  0.94  4.03  0.40  0.54  0.54  0.54  10.22  0.67  0.40  0.40 | 2.39E-06  2.31E-05  7.57E-05  0.0011  0.0015  0.0025  0.0071  0.0116  0.0147  0.0158  0.0183  0.0268  0.0356  0.0503  0.0573  0.0827  0.0827  0.0834  0.0883  0.0917  0.0917 |
| **Cellular Component** | | | | |
| GO:0005750  GO:0005751  GO:0005935  GO:0000131  GO:0005934  GO:0000145  GO:0005886  GO:0032153  GO:0005639  GO:0016586  GO:0005759  GO:0051286  GO:0070847  GO:0000164 | Mitochondrial respiratory chain complex III  Mitochondrial respiratory chain complex IV  Cellular bud neck  Incipient cellular bud site  Cellular bud tip  Exocyst  Plasma membrane  Cell division site  Integral component of nuclear inner membrane  RSC complex  Mitochondrial matrix  Cell tip  Core mediator complex  Protein phosphatase type 1 complex | 9  8  25  16  17  5  30  16  3  6  14  9  6  3 | 1.21  1.08  3.36  2.15  2.28  0.67  4.03  2.15  0.40  0.81  1.88  1.21  0.81  0.40 | 4.40E-06  7.59E-05  0.0014  0.0086  0.0097  0.0159  0.0226  0.0364  0.0518  0.0584  0.0693  0.0749  0.0909  0.0943 |

**S4 Table: List of enriched GO terms associated with down-regulated DEGs of acetate-grown *C. glabrata* cells**

| **GO accession** | **GO terms** | **Count** | **Frequency (%)** | **EASE score** |
| --- | --- | --- | --- | --- |
| **Biological Process** | | | | |
| GO:0008652  GO:0009097  GO:0006526  GO:0009098  GO:0009086  GO:0009088  GO:0019878  GO:0009082  GO:0006506  GO:0006207  GO:0009073  GO:0009423  GO:0055085  GO:0006696  GO:0006730  GO:0000103  GO:0002181  GO:0006096  GO:0019379  GO:0009090  GO:0006556  GO:0000056  GO:0006487 | Cellular amino acid biosynthetic process  Isoleucine biosynthetic process  Arginine biosynthetic process  Leucine biosynthetic process  Methionine biosynthetic process  Threonine biosynthetic process  Lysine biosynthetic process via aminoadipic acid  Branched-chain amino acid biosynthetic process  GPI anchor biosynthetic process  De novo pyrimidine nucleobase biosynthetic process  Aromatic amino acid family biosynthetic process  Chorismate biosynthetic process  Transmembrane transport  Ergosterol biosynthetic process  One-carbon metabolic process  Sulfate assimilation  Cytoplasmic translation  Glycolytic process  Sulfate assimilation, phosphoadenylyl sulfate reduction by phosphoadenylyl-sulfate reductase (thioredoxin)  Homoserine biosynthetic process  S-adenosylmethionine biosynthetic process  Ribosomal small subunit export from nucleus  Protein N-linked glycosylation | 8  6  7  5  7  5  5  5  8  5  4  4  13  9  5  4  8  6  3  3  3  5  7 | 1.09  0.82  0.96  0.68  0.96  0.68  0.68  0.68  1.09  0.68  0.55  0.55  1.78  1.23  0.68  0.55  1.09  0.82  0.41  0.41  0.41  0.68  0.96 | 2.29E-04  3.09E-04  4.73E-04  0.0018  0.0020  0.0049  0.0049  0.0049  0.0062  0.0101  0.0104  0.0104  0.0119  0.0190  0.0286  0.0414  0.0450  0.0511  0.0553  0.0553  0.0553  0.0793  0.0983 |
| **Molecular Function** | | | | |
| GO:0030170  GO:0051287  GO:0017176  GO:0004169  GO:0016791  GO:0005199  GO:0002161  GO:0015174  GO:0009922  GO:0102337  GO:0102338  GO:0102336  GO:0005215 | Pyridoxal phosphate binding  NAD binding  Phosphatidylinositol N-acetylglucosaminyltransferase activity  Dolichyl-phosphate-mannose-protein mannosyltransferase activity  Phosphatase activity  Structural constituent of cell wall  Aminoacyl-tRNA editing activity  Basic amino acid transmembrane transporter activity  Fatty acid elongase activity  3-oxo-cerotoyl-CoA synthase activity  3-oxo-lignoceronyl-CoA synthase activity  3-oxo-arachidoyl-CoA synthase activity  Transporter activity | 13  8  4  4  5  6  4  4  3  3  3  3  6 | 1.78  1.09  0.55  0.55  0.68  0.82  0.55  0.55  0.41  0.41  0.41  0.41  0.82 | 0.0027  0.0192  0.0291  0.0291  0.0378  0.0386  0.0516  0.0516  0.0647  0.0647  0.0647  0.0647  0.0884 |
| **Cellular Component** | | | | |
| GO:0016021  GO:0005789  GO:0005739  GO:0022626  GO:0009277  GO:0022627  GO:0017102  GO:0031415  GO:0030176  GO:0031207 | Integral component of membrane  Endoplasmic reticulum membrane  Mitochondrion  Cytosolic ribosome  Fungal-type cell wall  Cytosolic small ribosomal subunit  Methionyl glutamyl tRNA synthetase complex  NatA complex  Integral component of endoplasmic reticulum membrane  Sec62/Sec63 complex | 161  29  70  6  14  7  3  3  10  3 | 21.99  3.96  9.56  0.82  1.91  0.96  0.41  0.41  1.37  0.41 | 0.0000  0.0014  0.0067  0.0072  0.0365  0.0396  0.0532  0.0532  0.0550  0.0966 |

**S5 Table: List of enriched GO terms associated with DEPs of acetate-grown *C. glabrata***

Up-regulated proteins:

| **GO terms** | **Count** | **Frequency (%)** | **EASE score** |
| --- | --- | --- | --- |
| **Biological Process** | | | |
| Tricarboxylic acid cycle  Glyoxylate cycle  Aerobic respiration  ATP synthesis coupled proton transport  Mitochondrial electron transport, cytochrome c to oxygen  Mitochondrial electron transport, ubiquinol to cytochrome c  Fumarate metabolic process  Chronological cell aging | 9  3  6  4  3  3  2  3 | 5.4  1.8  3.6  2.4  1.8  1.8  1.2  1.8 | 2.70E-07  2.90E-03  6.40E-03  1.50E-02  2.50E-02  3.10E-02  6.20E-02  9.90E-02 |
| **Molecular Function** | | | |
| NAD binding  Proton-transporting ATP synthase activity, rotational mechanism  Cytochrome-c oxidase activity  Proton-transporting ATPase activity, rotational mechanism  Ubiquinol-cytochrome-c reductase activity  Threonine-type endopeptidase activity | 5  4  4  4  3  3 | 3.0  2.4  2.4  2.4  1.8  1.8 | 4.10E-03  6.10E-03  1.00E-02  2.90E-02  3.90E-02  8.80E-02 |
| **Cellular Component** | | | |
| Cytosol  Mitochondrial nucleoid  Mitochondrial respiratory chain complex IV  Mitochondrial respiratory chain complex III  Mitochondrial outer membrane  Mitochondrial matrix  Mitochondrion  Box H/ACA snoRNP complex | 45  6  4  4  5  6  18  2 | 26.8  3.6  2.4  2.4  3.0  3.6  10.7  1.2 | 1.60E-04  1.90E-04  2.80E-03  2.80E-03  2.00E-02  3.60E-02  5.50E-02  8.80E-02 |

Down-regulated proteins:

| **GO terms** | **Count** | **Frequency (%)** | **EASE score** |
| --- | --- | --- | --- |
| **Biological Process** | | | |
| rRNA export from nucleus | 2 | 4.3 | 5.90E-02 |
| **Molecular Function** | | | |
| Magnesium ion binding | 3 | 6.4 | 6.30E-02 |
